# Mr. Thomas William Nunn

**Published:** 1909-04-24

**Authors:** 


After a short illness, Mr. Thomas William Nunn, F.R.C.S., consulting surgeon to the Middlesex Hospital, died this week at the age of 84, at his country residence near Royston. Before being appointed to the surgical staff of Middlesex Hospital Mr. Nunn was surgeon to the Westminster Western Dispensary, and to the Central London Throat Hospital, and the London Hospital for Skin Diseases. He was formerly a lieutenant in the 3rd Middlesex Militia and honorary surgeon-major in the Volunteers. His chief contributions to medical literature dealt with the treatment of cancer, and in 1899 he published in book form the reports of 1,000 cases from the registers of Middlesex Hospital. Mr. Nunn was a past vice-president of the Pathological Society and a medical associate, and member of the teaching staff of the anatomical department of King's College. He obtained the membership of the Royal College of Surgeons of England in 1846, and the Fellowship in 1857.

